# The incidence, prevalence, and health burden of forearm fractures in China from 1992 to 2021 and forecasts for 2036

**DOI:** 10.3389/fpubh.2025.1566421

**Published:** 2025-06-24

**Authors:** Yuan Zeng, Minhua Hu, Zhiming Zhang, Jiayi Chen, Feifan Luan, ZhiSen Wu, Chenxiao Zheng

**Affiliations:** ^1^Zhongshan Hospital of Traditional Chinese Medicine Affiliated to Guangzhou University of Chinese Medicine, Zhongshan, China; ^2^Guangzhou University of Chinese Medicine, Guangzhou, China

**Keywords:** forearm fractures, China, incidence, years lived with disability, prevalence, GBD

## Abstract

**Background:**

Forearm fractures (Fracture of radius and/or ulna) are common worldwide and constitute a significant public-health burden. There is limited epidemiological data on radius and/or ulna fractures in the Chinese population. Using data from the Global Burden of Disease (GBD) 2021 study, this research reports the epidemiology and disease burden of forearm fractures in China, as well as projected trends for the next 15 years.

**Methods:**

Data on the incidence, prevalence, and years lived with disability (YLDs) for Fractures of the radius and ulna in mainland China from 1992 to 2021 were obtained from the 2021 GBD database. Joinpoint regression analysis was used to analyze epidemiological trends in incidence, prevalence, and age-standardized rates (ASIR, ASPR, ASYR) over the period from 1992 to 2021. The Bayesian Age-Period-Cohort (BAPC) model was employed to project trends in incidence and YLDs for radius and/or ulna fractures in mainland China from 2022 to 2036.

**Results:**

In 2021, there were 5,790,636 radius and/or ulna fractures in China, with 2,724,178 in males and 3,066,459 in females. The age-standardized incidence rate (ASIR) for radius and/or ulna fractures was 404.52 per 100,000, with 375.83 in males and 430.87 in females. The age-standardized years of disability (ASYR) was 2.55 years per 100,000, with 2.22 years in males and 2.82 years in females. From 1990 to 2021, both the ASIR and ASYR increased, with an average annual percent change (EAPC) of 0.09. The incidence of radius and/or ulna fractures peaked in males at ages 30–34, and in females at ages 50–54 and 65–69. Joinpoint regression analysis revealed a decline in incidence and prevalence from 2001 to 2005, followed by a significant increase from 2011 to 2021. Projected data suggests that the ASIR for radius and/or ulna fractures in males will rise from 369 per 100,000 to 374 per 100,000 from 2022 to 2029. This will be followed by a slight decline from 373 per 100,000 in 2030 to 368 per 100,000 in 2036, while the rate in females is expected to steadily increase from 427 per 100,000 in 2022 to 502 per 100,000 in 2036.

**Conclusion:**

In 2021, an estimated 5,790,636 radius and/or ulna fractures occurred in China. Prevalence and disability rates were higher among women than among men. After a decline between 2001 and 2005, the incidence, prevalence, and disability burden of these fractures rose sharply from 2011 to 2021. Projections indicate that incidence in men will fall slightly after 2030, whereas it will continue to rise in women. These findings provide critical evidence for shaping public-health policy, designing fracture-prevention programmes, and allocating healthcare resources in China.

## Introduction

In recent years, fractures have emerged as a major global public-health concern, and their prevalence continues to rise (1). Upper-extremity fractures (UEFs)—defined as fractures of the radius, ulna, clavicle, scapula, or humerus—rank among the three most common fracture types worldwide and constitute a large share of the overall fracture burden (2). Data from the Global Burden of Disease (GBD) 2019 study indicate that UEFs accounted for 38.7% of all fractures globally (2). Within this group, forearm fractures are the most frequent subtype and occur at similarly high rates in both high- and low-income settings (3, 4). For example, a 2019 analysis from the Middle East and North Africa reported more than 3.1 million radius/ulna fractures, far surpassing the counts for other upper-limb bones (5).

These fractures are frequently linked to trauma such as transport-related accidents or low-energy falls, particularly among osteoporotic women aged ≥ 50 years (6). As populations age and urbanisation accelerates, the burden of forearm fractures is projected to rise, driving up treatment costs and placing greater strain on healthcare systems (7, 8). Consequently, a detailed understanding of their epidemiology and risk factors is essential for policy-makers and orthopaedic surgeons who design cost-effective prevention and treatment strategies.

Although the epidemiology of upper-extremity fractures—including those of the ulna and radius—has been studied extensively, most investigations have centred on higher-income settings such as Europe, the United States, parts of the Middle East, and South Africa. Because cultural, economic, and demographic contexts differ widely, estimates derived from these regions cannot be applied directly elsewhere. China, the world’s most populous nation, has undergone rapid economic growth, urbanisation, and profound changes in social structure and lifestyle since the 1990s—developments that may have altered fracture incidence and its associated burden. At the same time, population ageing and the rising prevalence of osteoporosis suggest that the epidemiology of ulna and radius fractures could be following trajectories unique to China. Diet, physical-activity patterns, and access to healthcare are additional determinants. Several Chinese studies have described the epidemiology and economic impact of traumatic fractures, and a few have reported low-energy upper-extremity fractures in adults aged ≥ 50 years (9, 10). Yet these investigations were limited in age coverage, study period, or focus on forearm injuries; as a result, they do not capture the full burden of ulna and radius fractures across all age groups over the past three decades. To date, no comprehensive study has quantified the disease burden, temporal trends, and demographic patterns of these fractures in China. Such research is urgently needed to inform public-health policy, guide prevention and treatment strategies, and enrich comparative work in global fracture epidemiology.

The Global Burden of Disease (GBD) study—a worldwide collaboration that quantifies premature mortality, morbidity, and disability arising from 370 diseases and injuries in 204 countries and territories—provides extensive data on injuries and their risk factors (11). In this study, we focus on the epidemiology and disease burden of forearm fractures, specifically those involving the ulna and radius, in China, utilizing data from the GBD 2021 study. Furthermore, we project trends in the incidence and burden of these fractures over the next 15 years. Our objective is to provide a comprehensive analysis of the epidemiology and disease burden of radius/ulna fractures, offering critical insights to inform clinical practice and guide policy decisions.

## Materials and methods

### Data sources

The data used in this study were obtained from the GBD database, covering the period from 1990 to 2021. The GBD 2021 provides the most current epidemiological estimates on the burden of 371 diseases and injuries across 21 GBD regions and 204 countries and territories for the years 1990 to 2021 (12). These data are publicly available through the Global Health Data Exchange.^1^ The GBD 2021 classifies injuries based on both their causes and nature, identifying 30 mutually exclusive injury causes and 47 distinct injury properties. Fractures are classified as a specific type of injury, with further subcategories detailing fracture types, including those involving the radius and/or ulna. To obtain data on the incidence, prevalence, and years lived with disability (YLDs) for radius and/or ulna fractures, we used the query exchange tool of the GBD database. The data were sourced from various records, including vital registration data, insurance claims, health surveys, emergency department records, and hospital documentation. As this study did not involve human subjects, it was exempt from ethical review guidelines.

### Indexes

In this study, we report the age-standardized rates (ASRs) of incidence, prevalence, and YLDs due to forearm fractures in mainland China from 1990 to 2021. ASRs were calculated using the GBD world standard population to remove the effects of differing age structures and thus improve comparability across populations (13). All age-standardized rates, age-specific rates, along with their 95% uncertainty interval data (UI), are available from GBD. The incidence rate is defined as the number of new cases occurring within a specified period in a given population, expressed as the number of new cases per year divided by the population size at the midpoint of the year. The prevalence rate refers to the proportion of the population affected by radius and/or ulna fractures within a defined time frame and geographical region. YLDs measure the total years of healthy life lost due to disability from the time of fracture onset until death, quantifying the non-fatal health burden attributable to these injuries.

ASRs is a key metric that adjusts for the age composition of a population, facilitating comparisons across different populations or over time. This rate accounts for the distribution of various age groups, eliminating the confounding effect of age on statistical outcomes. To obtain the relevant data, we used the query tool in the GBD Results Visualization platform (VizHub—GBD Results) with the following parameters: GBD Estimate: Injuries by Nature; Measure: Years Lived with Disability, Prevalence, Incidence; Metric: Number, Percent, Rate; Injury: Fracture of Radius and/or Ulna; Cause: All Causes and Any Causes Under All Causes; Location: Global, China; Sex: Both; Period: 1990 to 2021. To ensure the accuracy and replicability of our findings, we adhered to the Guidelines for Accurate and Transparent Health Estimates Reporting (14).

### Joinpoint regression analysis

Joinpoint regression analysis is a statistical method used to identify points at which the linear trend of data related to fractures of the radius and/or ulna changes significantly. This model estimates changes in illness rates using the least squares method, which reduces the subjectivity often associated with traditional trend analyses based on linear assumptions. By calculating the sum of squared residuals between the estimated and actual values, the turning points where trend shifts occur are identified. Joinpoint software (version 4.9.1.0; National Cancer Institute, Rockville, MD, USA) was employed to construct this model. Additionally, we calculated the average annual percentage change (AAPC) and the annual percentage change (APC). The APC measures trend changes over a specific time period, while the AAPC provides a synthesized measure of overall change over time. An APC > 0 indicates year-on-year growth, whereas a negative APC suggests a decline. If the APC equals the AAPC, it implies no significant turning points in the trend. To characterize temporal trends in fracture burden, we applied Joinpoint regression analysis to assess epidemiological trends in incidence, prevalence, and age-standardized rates (ASIR, ASPR, ASYR) over the period from 1992 to 2021.

### Predicting incidence and YLDs

The Bayesian Age-Period-Cohort (BAPC) model is a statistical method derived from the Age-Period-Cohort (APC) framework, used to analyze and predict the effects of age, time period, and birth cohort on outcomes in demographic data (15). This model accounts for relationships between time series and demographic factors, such as changes in population structure, disease trends, and generational effects. Its considered factors include age, period, and cohort, but there is a linear relationship among the three factors, which may lead to the occurrence of nonunique parameter estimates. The BAPC model assumed that the effect of age, period, and cohort adjacent in time was analogous to obtain unique parameter estimates, and the obtained results are robust and reliable. In our study, we implemented the BAPC model using an integrated nested Laplace approximation (INLA) to estimate marginal posterior distributions and forecast the incidence and YLDs of radius and/or ulna fractures from 2022 through 2036. Age effects were stratified into consecutive 5-year intervals (0–4, 5–9, …, 90–94, 95 + years), while period effects were divided into 5-year segments covering both the historical window (1992–2021) and the projection window (2022–2036).

### Statistical analysis

In this study, statistical analyses were performed using R software (version 4.3.3). We employed ASIR, incidence rates, incidence numbers, YLD rates, and 95% UI to characterize the epidemiology and burden of radius and/or ulna fractures. The 95% UI was derived from the Bayesian-based model DisMod-MR 2.1 (16). To examine trends in ASIR and YLD rates over time, we used Joinpoint Regression (JPR) software (version 4.9.1.0) to construct the Joinpoint model and calculate the APC and AAPC from ASR for each year from 1990 to 2021. Finally, BAPC analysis was conducted using the BAPC and INLA packages in R software. A *p*-value of <0.05 was considered statistically significant.

## Results

### Descriptive analysis

In 2021, there were 5,790,636 cases of radius and/or ulna fractures in China (95% UI: 4,502,029–7,414,711), with 2,724,178 cases (95% UI: 2,163,261–3,401,242) in males and 3,066,459 cases (95% UI: 2,324,977–4,042,102) in females. Regarding prevalence, a total of 1,254,720 cases (95% UI: 1,052,076–1,505,777) were recorded in 2021, with 544,535 cases in males (95% UI: 451,436–653,979) and 710,185 cases in females (95% UI: 593,763–858,306). The total number of YLDs due to these fractures was approximately 42,524 YLDs (95% UI: 26,780–65,689), with 18,320 YLDs in males (95% UI: 11,283–28,778) and 24,203 YLDs in females (95% UI: 15,257–36,923). The ASIR for radius and/or ulna fractures was 404.52 per 100,000 population (95% UI: 317.08–516.51), with a lower rate of 375.83 per 100,000 in males (95% UI: 298.15–471.31) compared to 430.87 per 100,000 in females (95% UI: 329.47–566.73). The age-standardized YLD rate (ASYR) for these fractures was 2.55 per 100,000 population (95% UI: 1.56–4), with males exhibiting a rate of 2.22 per 100,000 (95% UI: 1.34–3.51), lower than the 2.82 per 100,000 rate in females (95% UI: 1.73–4.35) (Table 1). From 1990 to 2021, the estimated annual percentage change (EAPC) for ASIR was 0.09 (95% UI: 0.04–0.15) for males and 0.10 (95% UI: 0.03–0.14) for females. The EAPC for ASYR was 0.05 (95% UI: 0.01–0.09), with 0.06 (95% UI: 0.01–0.10) for males and 0.04 (95% UI: −0.01 to 0.09) for females.

**Table 1:** The incidence, prevalence and YLDs of forearm fractures in China and the temporal trends from 1990 to 2021.

Burden	Genders	Number in 2021 (95% UI)	ASR per 100,000 in 2021 (95% UI)	EAPC of ASR from 1990 to 2021 (95 %UI)
Incidence	Male	2,724,178 (2,163,261–3,401,242)	375.83 (298.15–471.31)	0.09 (0.03–0.14)
	Female	3,066,459 (2,324,977–4,042,102)	430.87 (329.47–566.73)	0.1 (0.03–0.16)
	Both Sexes	5,790,636 (4,502,029–7,414,711)	404.52 (317.08–516.51)	0.09 (0.04–0.15)
Prevalence	Male	544,535 (451,436–653,979)	67.57 (54.8–82.51)	0.07 (0.03–0.11)
	Female	710,185 (593,763–858,306)	85.07 (69.19–104.74)	0.06 (0.02–0.1)
	Both Sexes	1,254,720 (1,052,076–1,505,777)	77.15 (62.85–94.67)	0.06 (0.03–0.1)
YLDs	Male	18,320 (11,283–28,778)	2.22 (1.34–3.51)	0.06 (0.01–0.1)
	Female	24,203 (15,257–36,923)	2.82 (1.73–4.35)	0.04 (−0.01–0.09)
	Both Sexes	42,524 (26,780–65,689)	2.55 (1.56–4)	0.05 (0.01–0.09)

EAPC, estimated annual percentage change; UI, uncertainty interval; YLDs, years lived with disability; ASR, age-standardized rates.

**Figure 1:**
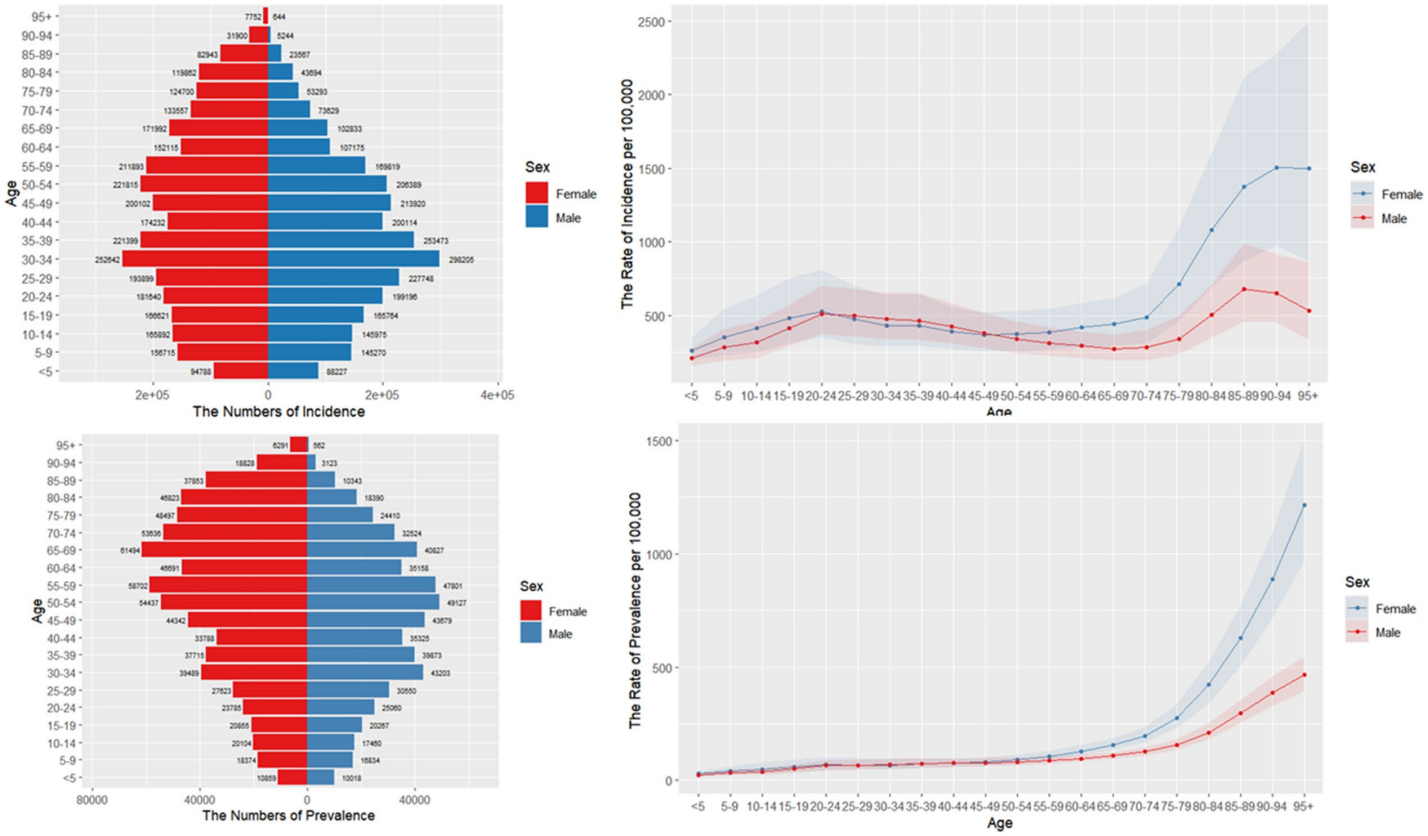
Age-specific numbers and age-standardized prevalence, incidence rates of forearm fractures in China, 2021.

Figure 1 illustrates the incidence, prevalence, and age-standardized rates of radius and/or ulna fractures across different age groups in 2021. The highest incidence of these fractures occurred in young adults aged 30–34 years for both sexes (Figure 1). In contrast, the peak prevalence in males was observed in the 65- to 69-year age group, while in females, it occurred in the 50- to 54-year age group (Figure 1). For individuals aged 25–49 years, the incidence of radius and/or ulna fractures was slightly higher in males than in females. However, this trend reversed in older age groups (Figure 1). After the age of 70, both the incidence and prevalence of these fractures increased rapidly in both sexes, with females exhibiting a higher incidence than males.

**Figure 2:**
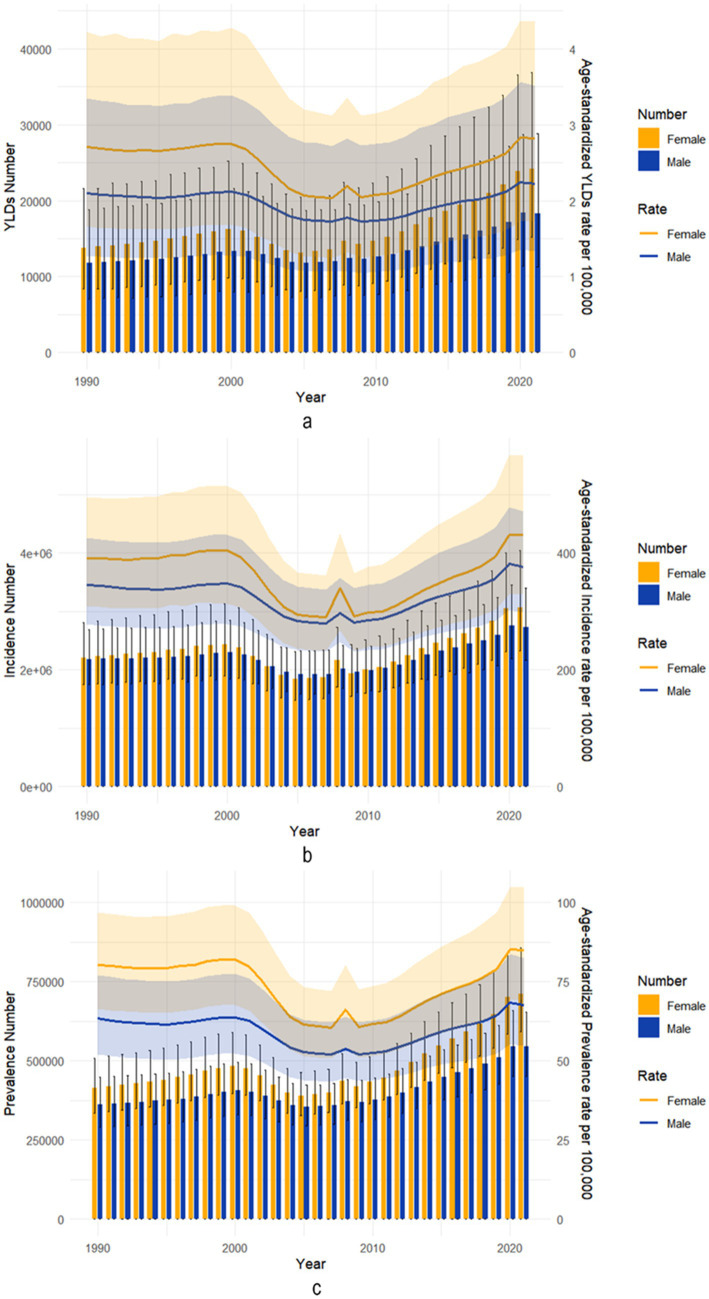
Trends in the all-age cases and age-standardized incidence, prevalence, and YLDs of forearm fractures sex from 1990 to 2021. **(a)** YLDs number and age-standardized rate. **(b)** Incidence number and age-standardized rate. **(c)** Prevalence number and age-standardized rate.

Figure 2 illustrates the number and age-standardized rates of gender-specific incidence, prevalence, and YLDs for radius and/or ulna fractures in China from 1990 to 2021. The age-standardized incidence, prevalence, and YLDs fluctuated over the years. The trends for all sexes and age groups were consistent, with little change observed from 1990 to 2000, followed by a gradual decline from 2000 to 2007. A sharp increase occurred in 2008, after which there was a steady rise from 2009 to 2021 (Figure 2).

### Joinpoint regression analysis

**Figure 3:**
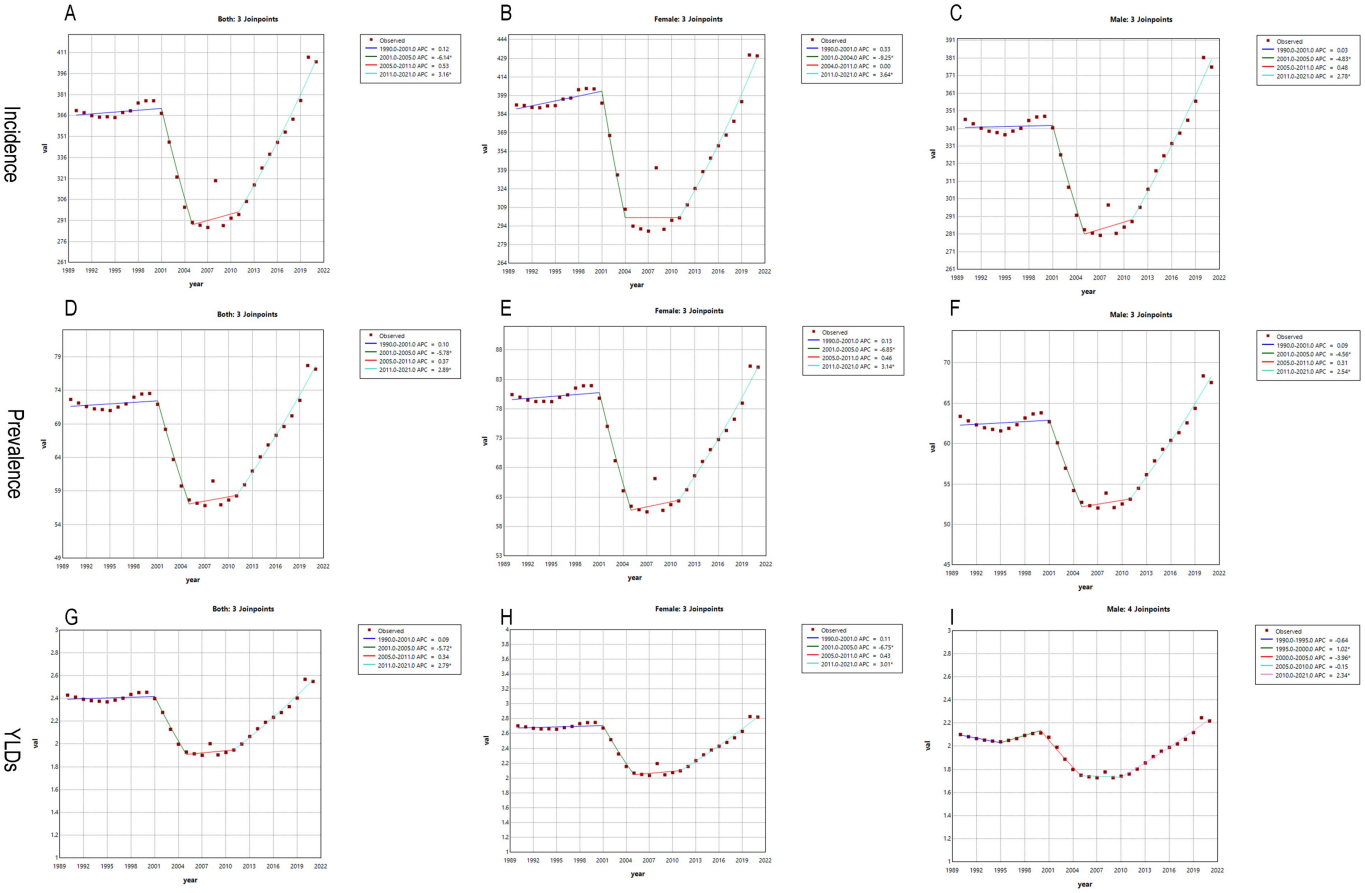
Joinpoint regression analysis: the APC in the incidence, prevalence, and YLDs of forearm fractures in China from 1992 to 2021. **(A,D,G)** Both; **(B,E,H**) Female; **(C,F,I)** Male; *Indicates that the APC is significantly different from zero at the alpha = 0.05.

We performed Joinpoint regression analysis on the ASIR, age-standardized prevalence rate (ASPR), and age-standardized ASYR for radius and/or ulna fractures from 1990 to 2021. The results are presented in Figure 3 and Table 1. As shown in Figure 3, from 2001 to 2005, the ASIR for both males (Figure 3C) and the combined sexes (Figure 3A) showed a gradual decline, with APC of −4.83 (95% CI: −6.95, −2.65) and −6.14 (95% CI: −9.37, −2.81), respectively (Supplementary Table 1). In females (Figure 3B), a similar decreasing trend was observed from 2001 to 2004, with an APC of −9.25 (95% CI: −17.35, −0.36) (Supplementary Table 1). However, from 2011 to 2021, all groups—males (APC = 2.77, 95% CI: 2.39, 3.15), females (APC = 3.64, 95% CI: 2.83, 4.44), and both sexes combined (APC = 3.16, 95% CI: 2.56, 3.76)—showed a significant increase in disease incidence (Figures 3A–C; Supplementary Table 1).

The trend for the ASPR mirrored that of the ASIR, with a consistent decline from 2001 to 2005 for all groups—males, females, and both sexes (Figures 3D–F)—with corresponding APCs of −4.56 (95% CI: −6.28, −2.80), −6.85 (95% CI: −9.40, −4.22), and −5.78 (95% CI: −7.87, −3.63), respectively (Supplementary Table 1). From 2011 to 2021, the trend reversed, showing an upward trajectory in ASPR for all groups, with APCs of 2.54 (95% CI: 2.24, 2.84) in males, 3.14 (95% CI: 2.67, 3.61) in females, and 2.89 (95% CI: 2.50, 3.28) for both sexes combined (Supplementary Table 1).

The ASYR for females and both sexes followed the ASPR trend, showing a decline each year from 2001 to 2005 (Figures 3G,H), with APCs of −6.75 (95% CI: −9.15, −4.29) and −5.72 (95% CI: −7.76, −3.64), respectively (Supplementary Table 1). Conversely, from 2011 to 2021, both groups exhibited an annual increase in ASYR, with APCs of 3.01 (95% CI: 2.61, 3.42) for females and 2.79 (95% CI: 2.45, 3.13) for both sexes combined (Supplementary Table 1). In contrast, the trend for ASYR in males showed a slight deviation, with an initial increase from 1995 to 2000 (APC = 1.02, 95% CI: 0.06, 1.99), followed by a decline from 2000 to 2005 (APC = −3.96, 95% CI: −4.86, −3.05), and a gradual increase again from 2010 to 2021 (APC = 2.34, 95% CI: 2.14, 2.55) (Figure 3I).

### Forecasting the incidence and YLDs of forearm fractures

**Figure 4:**
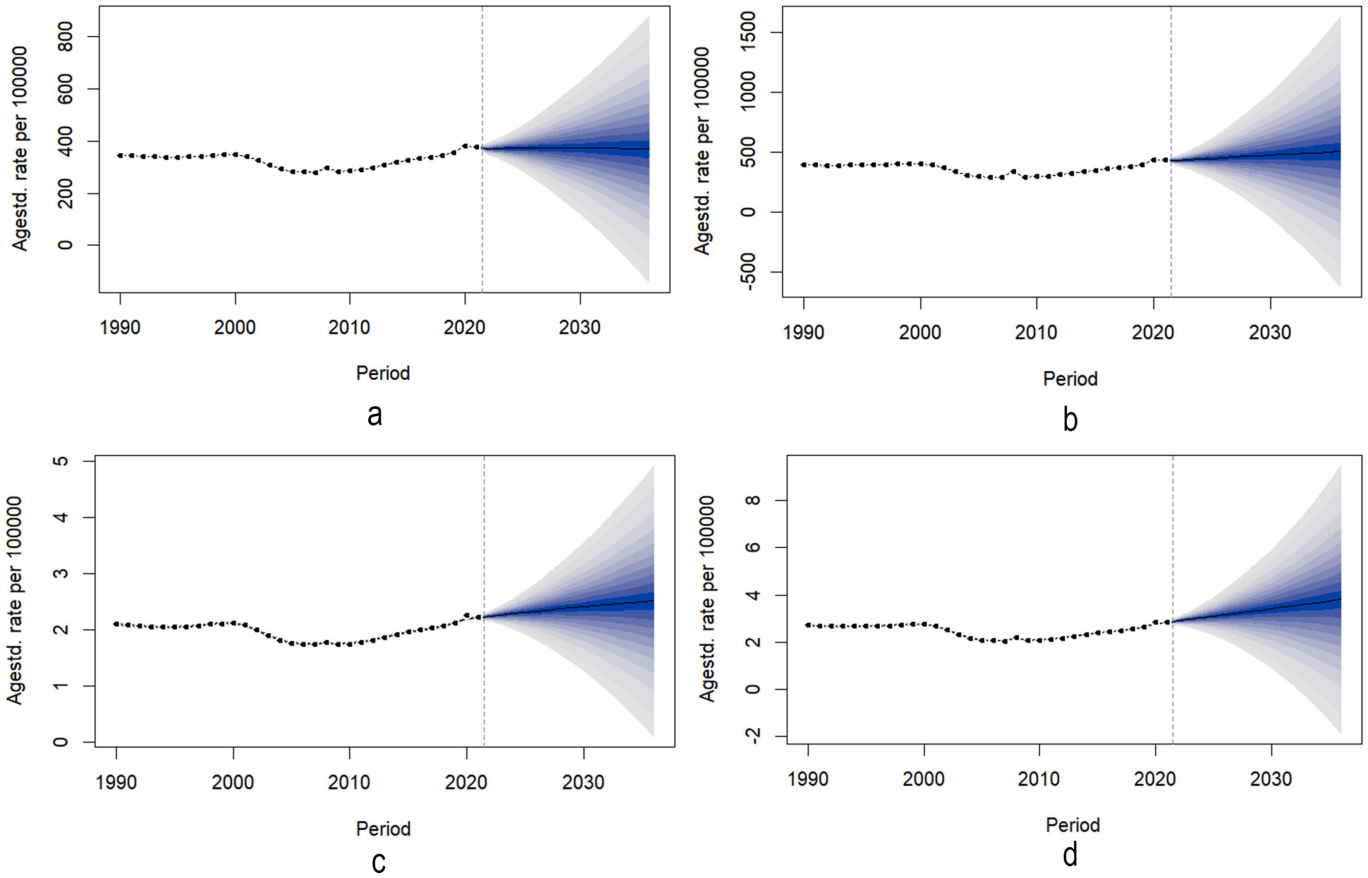
Predicted trends of the incidence and YLDs of Forearm fractures in China over 15 years. **(a)** ASR of incidence trends of male. **(b)** ASR of incidence trends of female. **(c)** ASR of YLDs trends of male. **(d)** ASR of YLDs trends of female.

Figures 4a,b illustrate the trends in the age-standardized incidence rate (ASIR) of radius and/or ulna fractures for males and females from 1990 to 2021, along with the projected trends for both sexes from 2022 to 2036. For Chinese males, the ASIR is predicted to show a gradual increase followed by a slow decrease from 2022 to 2035, with a projected inflection point in 2030. Specifically, from 2022 to 2029, the ASIR for males is expected to rise from 369 (95%UI: 358–380) per 100,000 to 374 (95%UI: 264–483) per 100,000 (Figure 4a, Supplementary Table 2). This will be followed by a slight decline from 373 (95%UI: 245–502) per 100,000 in 2030 to 368 (95%UI: 108–628) per 100,000 in 2036. In contrast, the ASIR for radius and/or ulna fractures in Chinese females is expected to steadily increase from 427 (95%UI: 407–448) per 100,000 in 2022 to 502 (95%UI: −70 to 1,075) per 100,000 in 2036 (Figure 4b, Supplementary Table 2).

Figures 4c,d show the changes in age-standardized years lived with disability (ASYR) due to radius and/or ulna fractures for males and females from 1990 to 2021, along with the projected trends from 2022 to 2036. For both males (Figure 4c) and females (Figure 4d), the ASYR is projected to gradually increase from 2022 to 2036. Specifically, the ASYR for males is expected to rise from 2.24 (95%UI: 2.19–2.29) per 100,000 in 2022 to 2.51 (95%UI: 1.28–3.74) per 100,000 in 2036, while for females, it is expected to increase from 2.91 (95%UI: 2.81–3.00) per 100,000 in 2022 to 3.80 (95%UI: 0.91–6.69) per 100,000 in 2036 (Supplementary Table 2). To assess robustness, we performed 10-year backcasting (2012–2021), comparing predicted rates with observed data, indicating reliable predictive performance.

## Discussion

In this study, we report the 2021 incidence, prevalence, and YLDs of radius and/or ulna fractures in China, characterize temporal trends from 1990 to 2021 using Joinpoint regression, and project incidence and DALYs through 2036 via Bayesian age–period–cohort modeling. To our knowledge, this is the first Chinese analysis to combine Joinpoint regression with APC methodology for examining long-term epidemiological trends in radius and ulna fractures.

Our results show that in 2021, the total incidence and prevalence of radius and/or ulna fractures in China were 5,790,636 and 1,254,720 cases, respectively. Both the ASIR and ASYR were higher in women than in men—430.87 versus 375.83 per 100,000 and 2.82 versus 2.22 per 100,000, respectively. This sex difference largely reflects the greater prevalence of osteoporosis among postmenopausal women, which heightens their fall risk with advancing age. Estrogen deficiency disrupts bone metabolism, reduces bone mineral density (BMD), and compromises bone microarchitecture; concomitantly, older women are more susceptible to falls owing to sarcopenia, impaired balance, and comorbidities (17). Furthermore, evidence indicates that Asian women—especially Chinese women—tend to have lower BMD than other ethnicities, attributable in part to smaller body frame and lower bone mineral content (18).

Our study also shows that the incidence of radius and/or ulna fractures peaked at ages 30–34 in 2021, while prevalence was highest at ages 65–69 in men and 50–54 in women. This pattern reflects a higher fracture risk among younger adults due to traumatic injuries from traffic accidents, sports, and occupational hazards, whereas middle-aged and older adults are more affected by degenerative bone changes and increased fall risk. As bone density declines with age, weakened bone strength and balance impairments make older individuals, particularly postmenopausal women, more susceptible to fractures.

We also observed a sharp increase in both the incidence and prevalence of radius and/or ulna fractures after age 70 in both sexes, consistent with the global trend of higher fracture risk in aging populations (19). As China, the world’s most populous country, undergoes rapid population aging, the older adult population (aged 65+) is expected to comprise nearly 14.2% of the total population by 2022 (20). Aging plays a significant role in fracture incidence, especially for common fractures like radius and/or ulna fractures, which are prevalent in older adults. The gradual decline in BMD and heightened fall risk with advancing age contribute to the increasing incidence of fractures in this group (19).

Our data also reveal that among individuals aged 25–49 years, the incidence of radius and/or ulna fractures is slightly higher in males than in females. This pattern aligns with the greater exposure of men to occupational hazards and high-risk recreational activities. Construction workers and industrial laborers in this age group—predominantly male in China’s workforce—face elevated risks of traumatic fractures due to frequent heavy lifting and machinery operation (21, 22). Furthermore, the higher frequency of young and middle-aged males participating in contact sports during leisure time, and the subsequent exposure to trauma, may also explain this gender disparity (23). However, this trend reverses in older age groups. After the age of 50, the incidence of radius and/or ulna fractures becomes slightly higher in females than in males. Both incidence and prevalence increase rapidly in both sexes, with females showing a higher incidence. This gender-based reversal in fracture risk may be attributed not only to biological factors, such as accelerated bone loss after menopause (24, 25), but also to behavioral and lifestyle influences. Insufficient physical activity is associated with decreased bone mineral density, while regular weight-bearing exercise enhances bone strength (26). Studies have shown that older women tend to engage less frequently in strength training or impact-loading activities compared to their male counterparts (27). The lack of exercise accelerates degenerative changes in the musculoskeletal system. Additionally, sociocultural factors—such as lower awareness or prioritization of osteoporosis prevention among certain female populations—may also contribute to differences in fracture susceptibility and osteoporosis management (28).

From 1990 to 2021, the ASIR, ASPR, and health burden (ASYR) of radius and/or ulna fractures in China exhibited fluctuating trends. Between 1990 and 2000, these indicators remained relatively stable. However, from 2000 to 2007, both incidence and prevalence gradually decreased. After 2008, these indicators saw a sharp rise and continued to increase through 2021. Several factors may explain these fluctuations, including improvements in the healthcare system, an aging population, and lifestyle changes. During 2000–2007, China’s healthcare system underwent significant improvements, with an increased focus on osteoporosis screening and treatment for the older adult, which helped reduce the risk of falls and fractures (29). Moreover, health education and lifestyle changes promoted during this period may have further contributed to the decline in fractures (30). The post-2008 surge in fracture burden may coincide with events surrounding the 2008 Beijing Olympics. Traffic accidents and sports injuries were frequently reported during the Games, which could have impacted fracture trends (31, 32). Statistics indicate that orthopedic trauma had the highest incidence among medical conditions in both competition and non-competition venues during the Olympics (33). From 2009 to 2021, the rising incidence and prevalence of radius and/or ulna fractures reflected China’s aging population. As the population ages, the incidence of fractures in older adults significantly exceeds that of younger groups (34). Furthermore, improvements in medical care have led to higher rates of diagnosis and treatment for fractures, contributing to the observed increase in fracture cases.

Our analysis of temporal trends in the disease burden of forearm fractures is consistent with findings from other countries experiencing similar demographic shifts or healthcare transitions. In Japan, for instance, a regional population-based study conducted in Hokkaido reported that the annual incidence of distal radius fractures ranged from 158.0 to 272.6 per 100,000 population between 2011 and 2020. Notably, the age-adjusted incidence in females showed a significant decreasing trend during this period (35). In contrast, India—another country undergoing comparable demographic changes—has seen an increasing burden of forearm fractures, particularly among younger, working-age populations. This upward trend is primarily attributed to occupational hazards and road traffic accidents (36). However, underreporting and disparities in healthcare access may obscure the true epidemiological burden, highlighting the urgent need for improved surveillance systems and preventive strategies. A similar trend has been observed in the Middle East and North Africa region, where upper-extremity fractures, especially in countries such as Saudi Arabia, Afghanistan, and Yemen, have shown a notable increase. Between 1990 and 2019, the ASIR of upper-extremity fractures in the region rose by 2.33%. Among all types of upper-extremity fractures, those of the radius and/or ulna exhibited the highest ASIR, reaching 505.32 per 100,000 population (4). These findings collectively emphasize the global variation in the epidemiology of radius and/or ulna fractures, shaped by demographic transitions, healthcare infrastructure, occupational risk factors, and the effectiveness of public health interventions.

Joinpoint regression analysis revealed that the ASIR and ASPR for radius and/or ulna fractures decreased between 2001 and 2005, but showed significant increases from 2011 to 2021. These trends are closely linked to China’s rapid economic growth and social structural changes. From 2001 to 2005, following China’s accession to the World Trade Organization (WTO), economic growth was steady, but infrastructure and public health development were still in their early stages. Despite accelerated urbanization, fracture prevention and treatment likely received less attention due to underdeveloped public health systems and limited health education. However, after 2011, as China’s economy entered a “new normal” phase, economic growth slowed, while urbanization accelerated. Changes in public lifestyles, including increased traffic accidents and sports injuries, may have contributed to the rise in fracture incidence (37, 38).

Given China’s aging population, the incidence and health burden of radius and/or ulna fractures are expected to continue rising in the future. Projections suggest that by 2036, the incidence of these fractures will keep increasing, particularly among women, with annual increases anticipated. This trend is driven by the rapid growth of China’s older adult population and the corresponding rise in fracture risk among older individuals. To address this issue, greater attention must be paid to fracture prevention in the older adult, particularly through early osteoporosis screening and interventions. Additionally, as China’s healthcare system continues to improve and urban healthcare resources expand, early diagnosis and treatment of fractures may help alleviate the health burden in the future (39). Although the BAPC model provides a reliable basis for trend forecasting, the accuracy of predictions extending to 2036 depends on the stability of underlying demographic and epidemiological trends. The effectiveness of health strategies (e.g., osteoporosis screening, anti-fracture therapies, or integrated geriatric care models) will directly influence the future trajectory of fracture incidence. Accelerating the implementation of targeted measures—such as nationwide bone-density screening or fracture liaison services for older adults—could substantially lower incidence rates. Conversely, if the urban–rural gap in healthcare access continues to widen, or if these interventions are delayed, any anticipated reductions may be undermined. Disruptions such as healthcare policy shifts, economic factors, or unforeseen health crises could alter the disease burden trajectory, introducing uncertainty into long-term forecasts. Furthermore, while the BAPC model offers a solid framework for long-term projections based on age, period, and cohort effects, the absence of external predictive variables—such as healthcare access, urbanization, and socioeconomic status—represents a limitation. Incorporating these exogenous factors through multivariate time series models or advanced machine learning approaches (e.g., ARIMA with exogenous inputs, LSTM networks) could further enhance the accuracy and generalizability of future projections.

Despite the valuable insights provided by the GBD 2021 database, this study has several inherent limitations. First, while the GBD database offers national-level estimates of disease burden, the indicators for incidence, prevalence, and disability related to radius and/or ulna fractures are aggregated at the national level, with no specific provincial or regional breakdowns. Significant disparities exist within China in terms of economic development and healthcare resource distribution, particularly between the eastern coastal regions, western regions, and urban versus rural areas. These regional disparities can result in substantial variations in the epidemiological characteristics and health burden of radius and/or ulna fractures. Therefore, national average data may not fully reflect these local differences, potentially leading to underestimates or overestimates of the disease burden in specific regions. Second, the lack of detailed data at the provincial and district levels limits the ability of this study to account for regional variations in radius and/or ulna fractures. This limitation restricts the capacity to provide precise policy recommendations for local governments and public health departments. To address this gap, future studies should incorporate more granular local data and conduct hierarchical analyses to better understand the specific conditions of each province and region, enabling the development of region-specific public health interventions. Additionally, the GBD database relies on indirect methods to estimate the burden of disease, and the accuracy and completeness of these estimates can be influenced by various uncontrollable factors. While the GBD program uses data from multiple high-quality sources, data collection and reporting may be less robust in remote areas, leading to underreporting or delays in data entry. Similarly, indirect estimation methods based on disability weights and hospital records might overlook minor fractures frequently managed in rural clinics or through traditional practices, which are less likely to be formally documented. Moreover, it is important to emphasize that the GBD estimates are modeled rather than directly derived from real-time hospital or clinical registry data. As such, they are subject to statistical assumptions, modeling strategies, and limitations in source data quality. These estimates may not capture the full clinical reality or reflect case confirmation through radiographic or clinical diagnostic criteria. The absence of direct validation using clinical or hospital-based records further limits the interpretability of our findings. In particular, without cross-verification from hospital information systems (HIS) or electronic medical records (EMRs), reporting bias or diagnostic misclassification may persist.

To improve the accuracy and reliability of these estimates, future studies could validate and refine the data through local surveys, population censuses, and hospital-based data collection. Efforts to integrate GBD estimates with empirical data from national or provincial health databases, such as discharge records, trauma registries, or insurance claim systems, could significantly enhance the robustness of epidemiological analyses. To gain a more comprehensive understanding of the epidemiological trends of radius and/or ulna fractures across China’s regions, future research should focus on obtaining more detailed provincial and regional data. This approach would not only provide a more accurate assessment of the disease burden but also support the development of region-specific healthcare policies, helping to ensure a more equitable allocation of healthcare resources and improve fracture prevention and management nationwide.

## Conclusion

This study analyzed the incidence, prevalence, and YLDs for radius and/or ulna fractures in China in 2021 using data from the GBD 2021 database. The findings revealed that, in 2021, there were 5,790,636 radius and/or ulna fractures in China, with both prevalence and disability rates being higher in women than in men. The burden of radius and/or ulna fractures declined from 2001 to 2005, but from 2011 to 2021, there was a significant increase in incidence, prevalence, and disability rates for both genders. Projections suggest that while the prevalence of fractures in males is expected to slightly decrease after 2030, the prevalence in females is projected to continue rising. These findings provide critical evidence for public health planning. The identification of high-risk age and sex groups can inform targeted prevention strategies, such as fall-prevention programs and osteoporosis screening, particularly for aging women. Additionally, our long-term projections offer essential data to anticipate future healthcare needs and optimize the allocation of medical resources. By revealing temporal trends and predicting future disease burden, this study supports the development of evidence-based health policies and fracture prevention programs tailored to the demographic profile of the Chinese population.

## Data Availability

The original contributions presented in the study are included in the article/Supplementary material, further inquiries can be directed to the corresponding author.

## Footnotes

^1^ https://ghdx.healthdata.org/gbd-2021/sources
